# LMTK3 inhibition affects microtubule stability

**DOI:** 10.1186/s12943-021-01345-3

**Published:** 2021-03-17

**Authors:** Chiara Cilibrasi, Angeliki Ditsiou, Athanasios Papakyriakou, George Mavridis, Murat Eravci, Justin Stebbing, Teresa Gagliano, Georgios Giamas

**Affiliations:** 1 Department of Biochemistry and Biomedicine, School of Life Sciences, University of Sussex, JMS Building, Falmer, Brighton, BN1 9QG UK; 2 National Centre for Scientific Research “Demokritos”, Institute of Biosciences and Applications, 15341 Athens, Greece; 3 Faculty of Medicine, Department of Surgery and Cancer, Imperial College, London, W12 0NN UK; 4 Department of Medical Science, University of Udine, 33100 Udine, Italy

**Keywords:** LMTK3, Kinase inhibitor, Breast cancer, Tubulin, NUSAP1

## Results and discussion

### C28 induces mitotic arrest in breast cancer cells

**Fig. 1:**
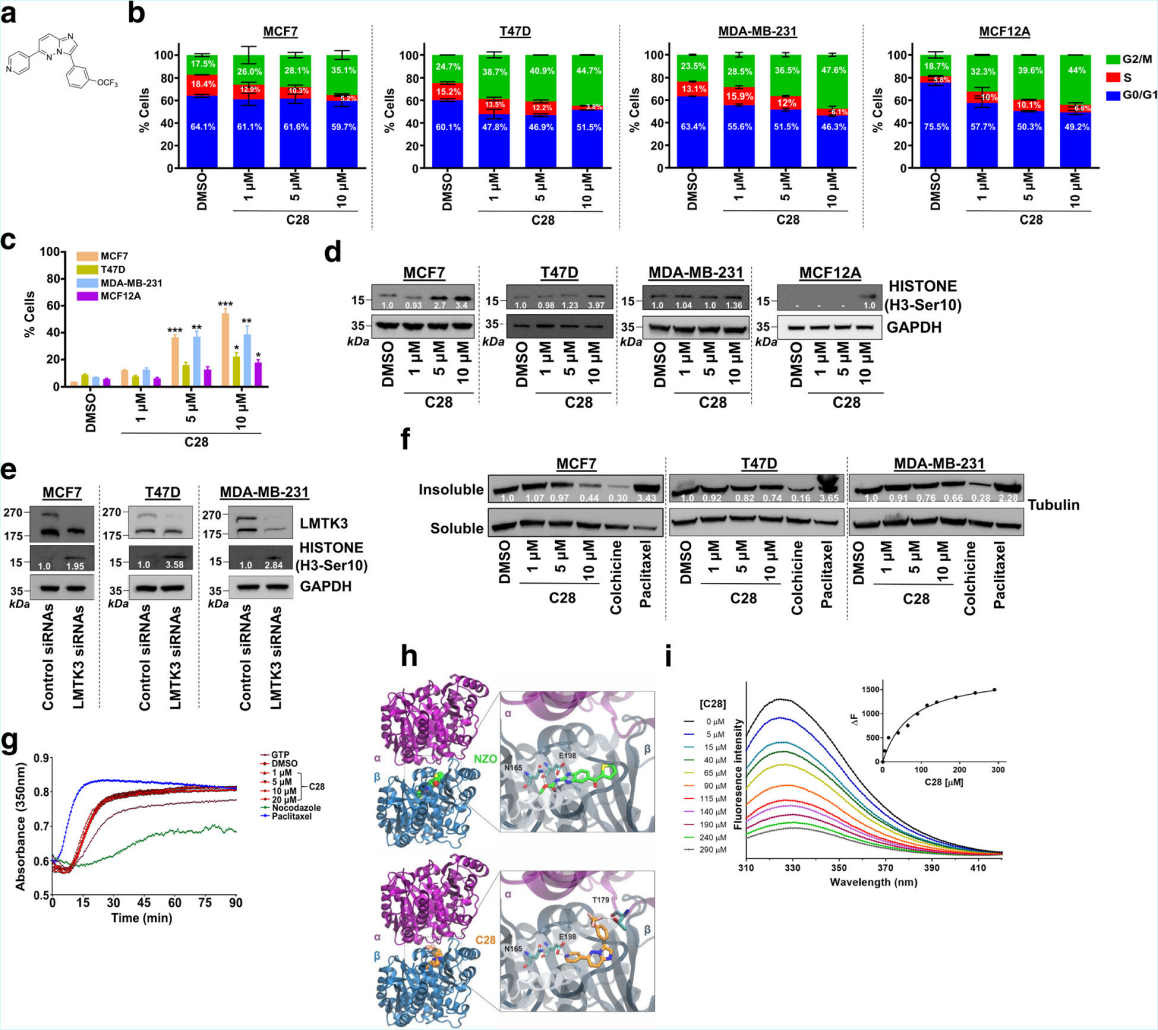
C28 induces the accumulation of mitotic cells and disrupts microtubule dynamics. **a** Chemical structure of C28. **b** Synchronized MCF7, T47D, MDA-MB-231 and MCF12A cell lines were analysed by flow cytometry (FACS) following treatment with increasing concentrations (0, 1, 5 and 10 μM) of C28 for 48 h. Percentages of cells in G0/G1, S and G2/M phase are indicated. Results are expressed as mean ± SEM. The experiment was performed two times. **c** MCF7, T47D, MDA-MB-231 and MCF12A cell lines were treated with increasing concentrations (0, 1, 5 and 10 μM) of C28 for 48 h and mitotic cells were counted. The mitotic index is represented as the percentage of mitotic cells over the total number of cells counted. The experiment was performed two times. ANOVA statistic test was performed using Prism 8 software. Results are expressed as mean ± SEM; * *P* < 0.05, ** *P* < 0.01. **d** Western blotting analysis of the phospho-histone H3 (Ser10) mitotic marker in MCF7, T47D, MDA-MB-231, MCF12A cell lines following treatment with increasing concentrations of C28 for 48 h. GADPH was used as loading control. Values represent the average of three experiments. **e** Western blotting analysis of the phospho-histone H3 (Ser10) mitotic marker in MCF7, T47D, MDA-MB-231 cell lines following siRNA silencing of LMTK3 for 72 h. GADPH was used as loading control. Values represent the average of three experiments. **f** MCF7, T47D and MDA-MB-231 cell lines were treated with increasing concentrations (0, 1, 5 and 10 μM) of C28 for 48 h. Cells were centrifuged to separate the insoluble (polymerized) and soluble (un-polymerized) tubulin, and fractions were analysed by western blotting. Colchicine (50 nM) or paclitaxel (50 nM) served as positive or negative control respectively. Values represent the ratio between the insoluble and soluble fractions and are the average of three experiments. **g** In vitro polymerization of bovine purified tubulin following incubation with increasing concentrations (0, 1, 5, 10 and 20 μM) of C28. Nocodazole (10 μM) or paclitaxel (10 μM) served as positive or negative controls respectively. The optical density (OD) was measured at 350 nm. **h** Molecular model of C28 bound to the colchicine site of tubulin in comparison with the X-ray crystal structure of tubulin complex with nocodazole (PDB ID: 5ca1) (29). The subunits α and β of tubulin are illustrated as purple and blue ribbons, respectively. Nocodazole (NZO) is shown with green C atoms, whereas C28 with orange C; all other atoms are coloured blue for N, red for O and pink for F. Potential hydrogen-bonding interactions between the ligands and key residues of tubulin are indicated with dashed lines. **i** Dose-dependent quenching of the intrinsic fluorescence of purified tubulin upon C28 binding. Fluorescence spectra of free tubulin 2 μΜ in PIPES buffer pH 6.9 (black line) and with increasing concentrations of C28 as indicated in the legend. Fluorescence was monitored at 25 °C in the range of 310 to 420 nm by excitation at 290 nm. Inset, is the change in the fluorescence intensity of tubulin heterodimers (ΔF) as a function of C28 concentration, from which the apparent dissociation constant (*K*_d_) was estimated to be 73 ± 15 μΜ by fitting to the equation described in the methods

Human lemur tyrosine kinase 3 (LMTK3) is a dual specificity kinase, whose oncogenic role has been well-established in different tumour types, supporting its potential as a therapeutic target [1]. Recently, using robust in vitro and cell-based screening- and selectivity- assays combined with biophysical analyses, we identified a selective small molecule ATP-competitive LMTK3 inhibitor (C28; Fig. 1a) that acts by degrading LMTK3 via the ubiquitin-proteasome pathway. C28 demonstrated effective anticancer effects in a variety of cancer cell lines and in in vivo breast cancer (BC) mouse models [2]. This potent, selective and cell-permeable inhibitor represents an effective tool to investigate and decipher the signalling pathways in which LMTK3 is implicated and progress with our translational research work.

In order to decipher the mechanism of action of C28, we initially performed flow cytometry (FACS) analysis and revealed that treatment with C28 leads to G2/M phase arrest of BC cells as well as MCF12A, a non-transformed breast cell line (Fig. 1b). This result was confirmed by evaluating the increased mitotic index (Fig. 1c) and by the upregulation of the mitotic marker phospho-histone H3 (Ser10) (Fig. 1d). As previously reported [2], prolonged exposure to C28 induced apoptosis of BC cells but had a low % of cell death (< 5%) in MCF12A, suggesting that non-transformed breast epithelial cells evade death while working on recuperating from cell cycle arrest (Fig. 1b). Similarly, silencing of LMTK3 led to an increase in phospho-histone H3 (Ser10) levels, suggesting a potential role of LMTK3 in G2/M transition (Fig. 1e), although no evident effects on cell cycle distribution were observed during the 72 h-period of siRNA treatment (data not shown), in accordance with previously published data [1].

### C28 interferes with tubulin polymerization and mitotic spindle organization

Bearing in mind the effects of C28 on cell cycle arrest and the induction of cell death, we investigated whether C28 impacts microtubule dynamics, which can in turn disturb the organization of the cytoskeleton and affect cell division [3]. Immunofluorescence of cells at the metaphase revealed that C28-treated cells present disrupted microtubule distribution and mitotic defects, including abnormal microtubule spindle organization and an altered chromosome condensation pattern (Supplementary Fig. S1a).

Following, the effect of C28 on microtubule stability was confirmed using a cell-based microtubule polymerization assay, where we observed a dose dependent decrease of the insoluble polymerized tubulin fraction. (Fig. 1f). Interestingly, the magnitude of C28 effects appeared to be cell-line dependent, suggesting the existence of different proteins and signalling pathways that may be implicated and affect this phenotype.

Considering the role of kinase inhibitors on microtubules [4, 5], we investigated the possibility that C28 is a direct tubulin-targeting agent by employing an in vitro tubulin polymerization assay. The effects on the assembly of purified porcine tubulin were evaluated by measuring the absorbance at 350 nm at 37 °C, using paclitaxel and nocodazole as comparative agents. As expected, paclitaxel promoted tubulin polymerization, while nocodazole inhibited it, as demonstrated by the increased fluorescence intensity (compared to the control) in the former and decrease in the latter. Contrariwise, incubation of tubulin with increasing concentrations of C28 at different time points had no effects on tubulin polymerization (Fig. 1g).

To examine whether C28 binds tubulin at the atomic level, we performed docking calculations of C28 in comparison with nocodazole, a reversible inhibitor of microtubule polymerization and a high-affinity ligand for the cancer-related kinases ABL, BRAF, c-KIT and MEK [6]. Our docking results using the high-resolution X-ray crystal structure of tubulin complex with nocodazole (PDB ID: 5ca1) revealed that C28 can be accommodated at the colchicine binding site of the β-subunit of tubulin (Fig. 1h). In particular, C28 is predicted to interact with both α and β subunits of the curved (unassembled) tubulin, but not as deep inside the β subunit as nocodazole. Compared with nocodazole that has been shown to bind β-tubulin via hydrogen-bonding interactions with Asn165 and Glu198 (Fig. 1h), C28 displayed a potential hydrogen bonding interaction with Glu198 and a halogen bond with Thr179 of the α-subunit. The free energy of binding to tubulin was estimated to be − 9.1 kcal/mol for C28 and − 10.4 kcal/mol for nocodazole, suggesting an approximately 10-fold lower affinity of C28 for tubulin.

The binding affinity of C28 to tubulin was further examined in vitro by monitoring the intrinsic tryptophan fluorescence of tubulin, a widely used method to determine the binding affinity of drugs for tubulin heterodimers [7]. Incubation of purified porcine tubulin with C28 revealed a concentration-dependent quenching of the fluorescence at 310–420 nm and the changes in the fluorescence intensity were fitted to a binding isotherm as previously described [7] (Fig. 1i). The apparent dissociation constant (*K*_d_) of C28 to purified tubulin heterodimers was estimated to be 73 ± 15 μΜ. For comparison, the binding affinity of nocodazole for purified tubulin was measured with apparent dissociation constants between 0.29 and 1.54 μΜ, depending on the specific tubulin isotype used. This result is in agreement with the lack of any observable effect on tubulin polymerization upon treatment with C28 concentrations of 1–20 μΜ in vitro (Fig. 1g).

Taken together, although C28 can potentially bind tubulin, its relatively low binding affinity to purified tubulin dimer and the lack of any observable effect on tubulin polymerization suggest that C28 does not confer its effects by direct inhibition of tubulin polymerization. Instead our data imply that C28 modulates LMTK3-regulated pathways linked to microtubule assembly, a result that can partly explain the previously observed universal cytotoxic effects of C28 on the NCI-60 cancer cell line panel [2].

### Pharmacological or genetic inhibition of LMTK3 downregulates NUSAP1 microtubule-associated protein

**Fig. 2:**
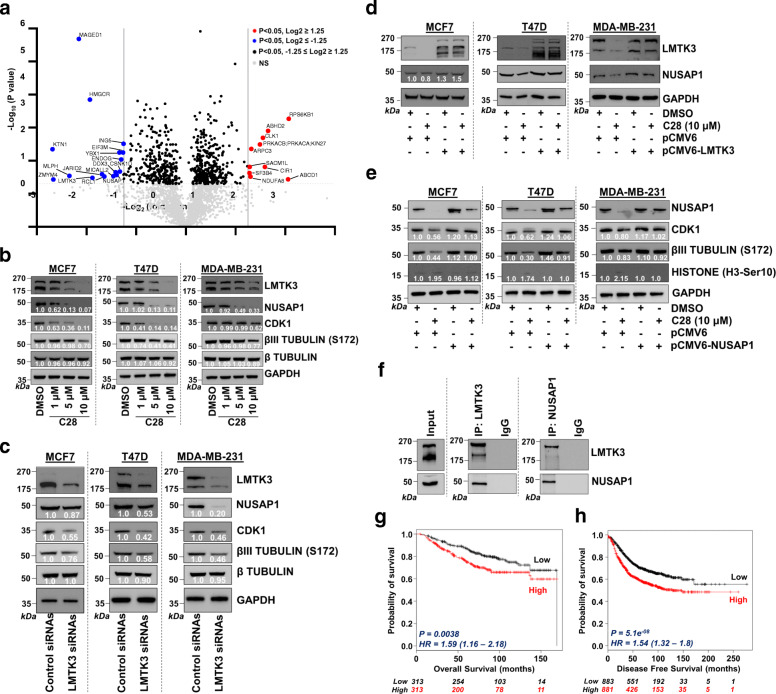
C28 decreases NUSAP1 protein levels. **a** Volcano plot of differentially expressed proteins following treatment with C28 in MCF7 cells stably overexpressing LMTK3. The plot illustrates the -Log_10_ *P*-value vs. the Log_2_ fold change of protein abundance in the presence of C28. The significance threshold (*P* = 0.05) is represented by a horizontal line. The two vertical lines (Log2 fold change of ≥1.5 and ≤ − 1.5) represent the cut-off values of interest. **b** Western blotting analysis of NUSAP1, CDK1, phospho-βIII tubulin (S172) and β tubulin in MCF7, T47D and MDA-MB-231 cell lines following treatment with increasing concentrations (0, 1, 5 and 10 μM) of C28 for 48 h. GADPH was used as loading control. Values represent the average of two experiments. **c** Western blotting of NUSAP1, CDK1, phospho-βIII tubulin (S172) and β tubulin in MCF7, T47D and MDA-MB-231 cell lines following inhibition (siRNA) of LMTK3. GADPH was used as loading control. Values represent the average of two experiments. **d** Western blotting showing the effects of LMTK3 overexpression, using a pCMV6-LMTK3 plasmid, on NUSAP1 protein levels in MCF7, T47D and MDA-MB-231 cell lines following 48 h pre-treatment with 10 μM C28. GADPH was used as loading control. Values represent the average of two experiments. **e** Western blotting analysis showing the effects of NUSAP1 overexpression, using a pCMV6-NUSAP1 plasmid, on CDK1, phospho-βIII tubulin (S172) and phospho-histone H3 (Ser10) in MCF7, T47D and MDA-MB-231 cell lines following 48 h pre-treatment with 10 μM C28. GADPH was used as loading control. Values represent the average of two experiments. **f** LMTK3 or NUSAP1 were immunoprecipitated from MCF7 cells stably overexpressing LMTK3, and the complexes were immunoblotted for LMTK3 and NUSAP1. Western blots for the respective proteins in whole cell lysates (input) were also performed. **g** Kaplan-Meier plots (http://kmplot.com/) demonstrating the association of the mean expression of LMTK3 and NUSAP1 with overall survival in 626 BC patients. HR, hazard ratio. **h** Kaplan-Meier plots (http://kmplot.com/) demonstrating the association of the mean expression of LMTK3 and NUSAP1 with disease free survival in 1764 BC patients

In an attempt to decipher the signalling pathways via which C28 confers its effects on microtubules assembly, we used a tandem mass tagging (TMT) quantitative proteomic approach and uncovered the C28-mediated global proteomic alterations in BC cells (Fig. 2a). Following quantile normalization of TMT label intensities for each channel, 2852 distinguishable and unambiguous proteins were identified with a minimum of one unique peptide with a false discovery rate (FDR) of 1% (Supplementary Table S1). As anticipated, LMTK3 was amongst the downregulated proteins following treatment with C28 (Fig. 2a).

Interestingly, amongst the most significant C28-modulated hits was NUSAP1 (*P* < 0.05 and Log2 fold change of ≥|1.25|), a microtubule associated protein participating in mitotic spindle organization [8, 9], whose role has been closely associated with tumour progression, chemoresistance, and poor prognosis in many tumours including BC [10, 11]. The effects of C28 on NUSAP1 were validated in additional BC cell lines (Fig. 2b). In addition, to confirm the involvement of NUSAP1 in the C28-mediated effect on microtubule stability and cell cycle arrest, we investigated the levels of cyclin-dependent kinase 1 (CDK1), a previously described NUSAP1-regulated protein [12], and phospho-βIII tubulin (S172). This latter phosphorylation is catalysed by CDK1 and has been shown to influence microtubule dynamics by affecting their polymerization [13, 14] (Fig. 2b) To corroborate that the observed effects are due to the selective C28-mediated LMTK3 inhibition, we silenced LMTK3 (siRNA) and saw a downregulation of NUSAP1, CDK1, and phospho-βIII tubulin (S172) (Fig. 2c). To further establish that this was an LMTK3/NUSAP1-mediated effect, we restored LMTK3 levels following C28 treatment and detected a rescue of NUSAP1 levels (Fig. 2d). In addition, a recovery of CDK1, phospho-βIII tubulin (S172) and phospho-histone H3 (Ser10) levels were observed after restoring NUSAP1 following C28 treatment (Fig. 2e). Altogether, these data confirm that C28 affects microtubule dynamics via an LMTK3 and NUSAP1-regulated pathway. Moreover, following immunoprecipitation experiments, an endogenous interaction of LMTK3 with NUSAP1 was discovered (Fig. 2f), suggesting, as previously reported [15], that LMTK3 may function as a scaffold protein able to interact with and stabilize NUSAP1.

Finally, we investigated the association between mRNA levels of LMTK3 and NUSAP1 with survival. Our analysis revealed that high expression of LMTK3 and NUSAP1 correlates with shorter overall survival (Fig. 2g) and disease-free survival (Fig. 2h), indicating a plausible cooperation of LMTK3 and NUSAP1 in BC progression.

## Conclusions

In the present study, we further demonstrated that C28 exerts its antitumor functions by degrading LMTK3 [2], which leads to a NUSAP1-mediated microtubule instability with subsequent cell-cycle arrest and cell death. Moreover, downregulation of NUSAP1-downstream proteins involved in cell cycle regulation and microtubule stability (CDK1 and phospho-βIII tubulin), after pharmacological and genetic LMTK3 inhibition, support this hypothesis (Supplementary Fig. S1b). Although we cannot rule out the possibility that the anticancer activity of C28 is also due to off-target effects, our LMTK3 and NUSAP1 recovery studies support that the observed results on microtubule stability are predominantly mediated via LMTK3 inhibition. In addition, the identification of NUSAP1 as a new interacting partner of LMTK3 suggests a direct interplay between LMTK3 and NUSAP1 pathways in the regulation of microtubule stability and validates the LMTK3 scaffolding properties, which can eventually contribute to tumorigenesis by enhancing signalling complexity [15]. Furthermore, our clinical data imply an association between NUSAP1 and LMTK3 in BC progression as shown by their unfavourable prognosis.

Combined, our findings propose that the pre-clinical therapeutic advantage of C28 stems from its effect on the LMTK3-targeted pathways linked to microtubule organization, acting differently from the established role of chemotherapeutic agents including vinca-alkaloids [16], taxanes [16] or eribulin [17], which confer their cytotoxicity via their interactions with tubulin causing disruption of microtubule function. The clinical use of microtubule-targeting drugs as anticancer drugs is well-established [18], while the ability of standard microtubule agents (i.e. nocodazole) to modulate the activities of certain kinases has also been reported [6]. Taken together, the development and design of multifunctional inhibitors can provide new, promising approaches for cancer treatment.

## Supplementary Information

**Additional file 1: Supplementary** **Table S1.** Identified proteins and comparative quantification of TMT label intensities.

**Additional file 2: Supplementary Figure S1.** Effects of C28 on microtubule organization in BC and non-transformed breast cell lines and schematic model depicting the proposed mechanism of action of C28 inhibitor. (a) MCF7, T47D, MDA-MB-231 and MCF12A cells were treated with 10 μM of C28 for 48 h. Cells were fixed and stained with anti-α-tubulin antibody (green) while the nuclear DNA was stained by DAPI (blue). Representative confocal microscopy images of mitotic phase cells are shown. Scale bar, 5 μm. (b) C28 binds to LMTK3 promoting its proteasome-mediated degradation. Downregulation of LMTK3 leads to a decrease in NUSAP1 and downstream proteins CDK1 and phospho-β III tubulin (S172), resulting in cell cycle arrest, abnormal spindles and microtubules instability.

**Additional file 3.** Supplementary Materials and Methods.

## Data Availability

The mass spectrometry proteomics data have been deposited to the ProteomeXchange Consortium via the PRIDE [19] partner repository with the dataset identifier PXD024644 and 10.6019/PXD024644. All the other data are available from the authors upon reasonable request.

## Abbreviations

LMTK3: Lemur tyrosine kinase 3
BC: Breast cancer
*K*_d_: Dissociation constant
NCI: National cancer institute
ABL: Tyrosine-protein kinase ABL1
BRAF: Proto-oncogene BRAF
c-KIT: Proto-oncogene c-KIT
MEK: Mitogen-Activated Protein Kinase Kinase 1
TMT: Tandem mass tagging
NUSAP1: Nucleolar and spindle associated protein 1
