# Correction to: LMTK3 inhibition affects microtubule Stability

**DOI:** 10.1186/s12943-021-01353-3

**Published:** 2021-04-09

**Authors:** Chiara Cilibrasi, Angeliki Ditsiou, Athanasios Papakyriakou, George Mavridis, Murat Eravci, Justin Stebbing, Teresa Gagliano, Georgios Giamas

**Affiliations:** 1 Department of Biochemistry and Biomedicine, School of Life Sciences, University of Sussex, JMS Building, Falmer, Brighton, BN1 9QG UK; 2 National Centre for Scientific Research “Demokritos”, Institute of Biosciences and Applications, 15341 Athens, Greece; 3 Department of Surgery and Cancer, Faculty of Medicine, Imperial College, W12 0NN, London, UK; 4 Department of Medical Science, University of Udine, 33100 Udine, Italy

**Correction to: Mol Cancer 20, 53 (2021)**

**https://doi.org/10.1186/s12943-021-01345-3**

Following the publication of the original article [1], the authors noticed errors on the figures introduced during the production step. Below are the errors:

Fig. 2a: The labels for the y- and x-axes have been translocated upwards and need to be realigned with the axes. The x-axis should read: -Log_2_ (fold change).

Fig. 2d: the quantification values in the NUSAP1 blots, for T47D and MDA-MB-231 cells, are no longer visible.

The original article has been corrected.

**Fig. 2:**
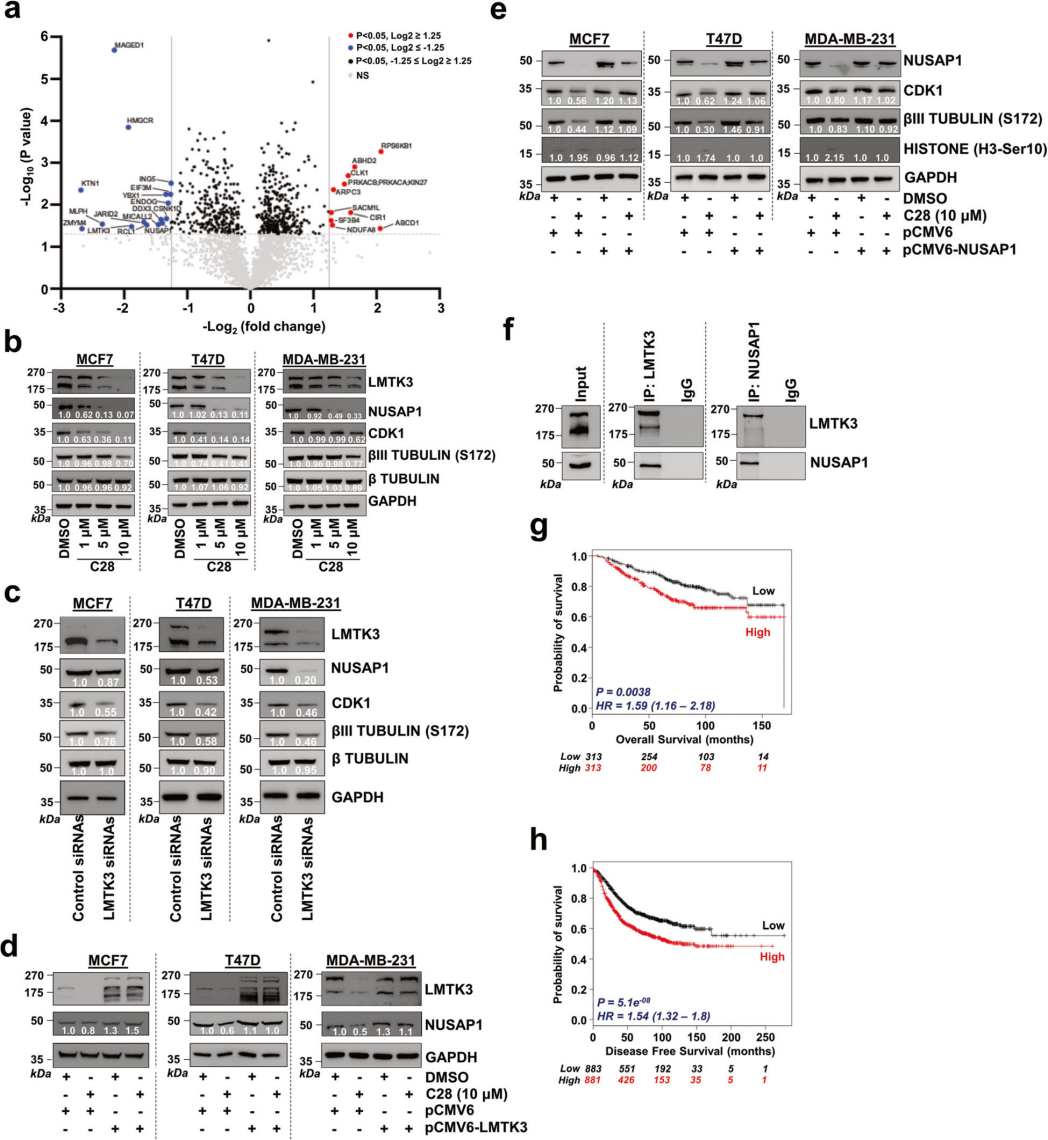
C28 decreases NUSAP1 protein levels. **a** Volcano plot of differentially expressed proteins following treatment with C28 in MCF7 cells stably overexpressing LMTK3. The plot illustrates the -Log10 *P*-value vs. the Log2 fold change of protein abundance in the presence of C28. The significance threshold (*P* = 0.05) is represented by a horizontal line. The two vertical lines (Log2 fold change of ≥1.5 and≤−1.5) represent the cut-off values of interest. **b** Western blotting analysis of NUSAP1, CDK1, phospho-βIII tubulin (S172) and β tubulin in MCF7, T47D and MDA-MB-231 cell lines following treatment with increasing concentrations (0, 1, 5 and 10 μM) of C28 for 48 h. GADPH was used as loading control. Values represent the average of two experiments. **c** Western blotting of NUSAP1, CDK1, phospho-βIII tubulin (S172) and β tubulin in MCF7, T47D and MDA-MB-231 cell lines following inhibition (siRNA) of LMTK3. GADPH was used as loading control. Values represent the average of two experiments. **d** Western blotting showing the effects of LMTK3 overexpression, using a pCMV6-LMTK3 plasmid, on NUSAP1 protein levels in MCF7, T47D and MDA-MB-231 cell lines following 48 h pre-treatment with 10 μM C28. GADPH was used as loading control. Values represent the average of two experiments. **e** Western blotting analysis showing the effects of NUSAP1 overexpression, using a pCMV6-NUSAP1 plasmid, on CDK1, phospho-βIII tubulin (S172) and phospho-histone H3 (Ser10) in MCF7, T47D and MDA-MB-231 cell lines following 48 h pre-treatment with 10 μM C28. GADPH was used as loading control. Values represent the average of two experiments. **f** LMTK3 or NUSAP1 were immunoprecipitated from MCF7 cells stably overexpressing LMTK3, and the complexes were immunoblotted for LMTK3 and NUSAP1. Western blots for the respective proteins in whole cell lysates (input) were also performed. **g** Kaplan-Meier plots (http://kmplot.com/) demonstrating the association of the mean expression of LMTK3 and NUSAP1 with overall survival in 626 BC patients. HR, hazard ratio. **h** Kaplan-Meier plots (http://kmplot.com/) demonstrating the association of the mean expression of LMTK3 and NUSAP1 with disease free survival in 1764 BC patients
