# Supplementary figures and images for: LMTK3 inhibition affects microtubule stability
Source: Mol Cancer. 2021 Mar 17;20:53. doi: 10.1186/s12943-021-01345-3 (PMC7968321; doi:10.1186/s12943-021-01345-3)

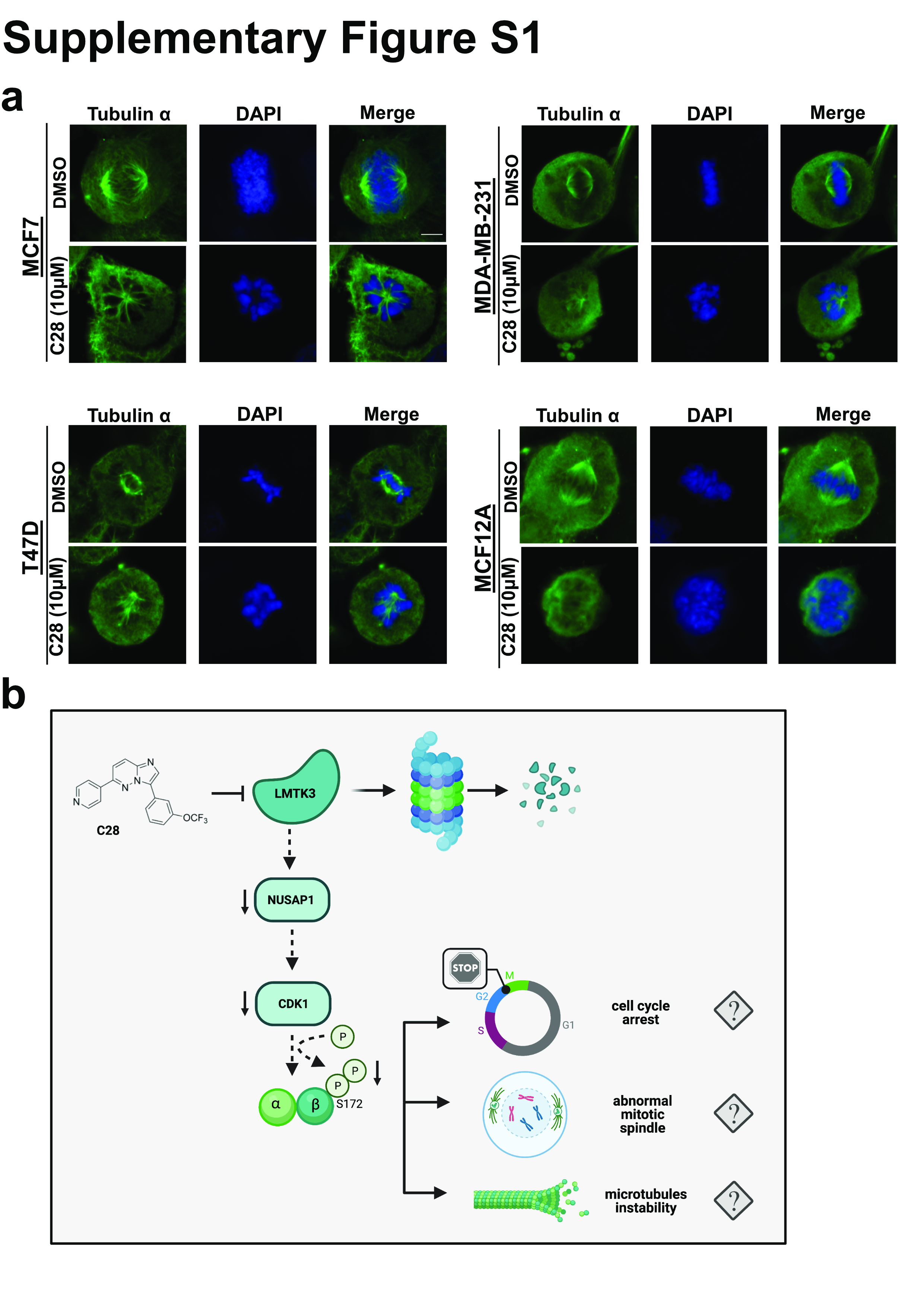

Supplement: Supplementary file 2 — Additional file 2: Supplementary Figure S1. Effects of C28 on microtubule organization in BC and non-transformed breast cell lines and schematic model depicting the proposed mechanism of action of C28 inhibitor. (a) MCF7, T47D, MDA-MB-231 and MCF12A cells were treated with 10 μM of C28 for 48 h. Cells were fixed and stained with anti-α-tubulin antibody (green) while the nuclear DNA was stained by DAPI (blue). Representative confocal microscopy images of mitotic phase cells are shown. Scale bar, 5 μm. (b) C28 binds to LMTK3 promoting its proteasome-mediated degradation. Downregulation of LMTK3 leads to a decrease in NUSAP1 and downstream proteins CDK1 and phospho-β III tubulin (S172), resulting in cell cycle arrest, abnormal spindles and microtubules instability. [file 12943_2021_1345_MOESM2_ESM.docx]
