# Supplementary material for: LMTK3 inhibition affects microtubule stability
Source: Mol Cancer. 2021 Mar 17;20:53. doi: 10.1186/s12943-021-01345-3 (PMC7968321; doi:10.1186/s12943-021-01345-3)
Supplement: Supplementary file 3 — Additional file 3. Supplementary Materials and Methods. [file 12943_2021_1345_MOESM3_ESM.docx]

**Supplementary Materials and Methods**

**Reagents**

Paclitaxel (cat. no. T1912-1MG) and colchicine (cat. no. C9754-100MG) were purchased from Sigma Aldrich and resuspended in DMSO (Millipore, cat. no. D/4125/PB08). β tubulin (cat. no. 86298) and phospho-Histone H3 (Ser10) (cat. no. 3377) antibodies as well as anti-rabbit IgG (1:5000, cat. no. 7074P2) and anti-mouse IgG (1:5000, cat. no. 7076P2) HRP-linked antibodies were purchased from Cell Signaling. CDK1 (cat. no. STJ23048) and NUSAP1 (anti-ANKT) (1:1000, cat. no. STJ91598) antibodies were purchased from St John’s Labs. Phospho-βIII tubulin S172 antibody was purchased from Abcam (cat. no. ab76286). GAPDH (1 μg/mL, cat. no. 39-8600) and Alexa Flour®-488 goat anti-mouse (cat. no. A11029) secondary antibody were purchased from Thermo Fisher Scientific, while α-tubulin (0.5 μg/mL or 1:100, cat. no. A01410-100) was purchased from GenScript. A Sigma Aldrich antibody (1:500, cat no. WH0114783M2) was used to detect total LMTK3. A pCMV6-LMTK3 overexpressing plasmid (cat. no. RC223140), the pCMV6 empty vector (cat. no. PS100001) and the pCMV6-NUSAP1 plasmid (cat. no. RG219159) were purchased from Origene. Purified porcine tubulin was purchased from Cytoskeleton Inc. (cat. no. T240). All the other reagents, if not otherwise specified, were purchased from Thermo Fisher Scientific.

**Cell lines**

MCF7, T47D, MDA-MB-231 and MCF12A cell lines were purchased from ATCC. MCF7 and MDA-MB-231 cells were maintained in low glucose DMEM (Sigma Aldrich, cat. no. D6046-500ML) supplemented with 10% FBS (Sigma Aldrich, cat. no. F7524-500ML) and 1% Penicillin/Streptomycin (Sigma-Aldrich, cat. no. P0781-100ML). T47D cells were maintained in RPMI-1640 medium (Sigma Aldrich, cat. no. R5886-500ML) supplemented with 10% FBS (Sigma Aldrich, cat. no. F7524-500ML) and 1% L-glutamine/Penicillin/Streptomycin solution (Sigma-Aldrich, cat. no. G1146-100ML). MCF12A cells were maintained in Cascade Biologics medium 171 (Gibco, cat. no. 171 M-171-500) supplemented with 10% FBS (Sigma Aldrich, cat. no. F7524-500ML), 1% L-Glutamine/Penicillin/Streptomycin (Sigma Aldrich, cat. no. G1146-100ML), 1% mammary epithelial growth supplement (Thermo Fisher Scientific, cat. no. S0155) and 100 ng/mL cholera toxin from Vibrio cholerae (Sigma Aldrich, cat. no. C8052-5MG).

**Western blotting**

Protein lysates were extracted using RIPA buffer (Sigma Aldrich, cat. no. R0278-50ML) including fresh protease and phosphatase inhibitors (Roche Diagnostics GmbH, cat. no. 11697498001 & cat. no. 4906845001) and standard western blotting protocol was performed. Briefly, protein concentration of the lysates was determined using the Pierce BCA protein assay kit (ThermoFisher Scientific, cat. no. 23227) and 30-50 μg of protein extract was resolved on SDS/PAGE gels (GenScript, cat. no. M42012 & cat. no. M00653). Proteins were then transferred onto a nitrocellulose blotting membrane (Thermo Fisher Scientific, cat. no. IB23001) using the iBlot 2 dry blotting system (Thermo Fisher Scientific, cat. no. IB21001). The membranes were blocked in TBS containing 0.1% (v/v) Tween 20 and 5% (w/v) non-fat milk or 5% (w/v) BSA (VWR Life Science, cat. no. 421501J) for 1 hour before being incubated with the primary antibodies overnight at 4°C. HRP-conjugated secondary anti-rabbit (1:5000, Cell Signaling, cat. no. 7074P2) or anti-mouse (1:5000, Cell Signaling, cat. no. 7076P2) antibodies were used. Chemiluminescent detection was performed using the SuperSignal West Pico PLUS Chemiluminescent Substrate (Thermo Fisher Scientific, cat. no. 34577). Emission was captured using the UVP ChemStudio Imaging Systems (Analityk jena).

**Immunofluorescence staining**

Cells grown on glass coverslips were fixed in 4% formaldehyde in cytoskeleton buffer (10mM MES pH 6.1, 138mM KCl, 3mM MgCl, 2mM EGTA, 0.32M sucrose) for 15 minutes, washed in PBS and permeabilized in 0,1% NP40. Coverslips were then incubated for 90 minutes at room temperature with α-tubulin (1:100, Genscript, cat. no. A01410-100) primary antibody diluted in 2% BSA. Cells were washed and probed with Alexa Flour®-488 goat anti-mouse (Thermo Fisher Scientific, cat. no. A11029) secondary antibody at room temperature for 60 minutes. After washing, coverslips were mounted onto glass slides with the Slowfade Gold antifade reagent with DAPI (Thermo Fisher Scientific, cat. no. S36942). Images were acquired through a 63x oil immersion lens on Zeiss LSM880 confocal microscope equipped with a CCD camera.

**Cell cycle analysis**

Cells were synchronized by serum starvation and then treated with increasing concentrations of C28 for 48 h in complete medium. After collection, the DNA was labelled with propidium iodide using the BD Cycletest Plus DNA Reagent Kit (BD Biosciences, cat. no. 340242) following the manufacturer’s instructions. The BD Accuri C6 flow cytometer (BD Biosciences) was used.

**Mitotic index assay**

Cells grown on glass coverslips were fixed in 4% paraformaldehyde for 15 minutes, washed in PBS and incubated with 0.3% (v/v) Triton X-100, 3% BSA in PBS for 30 minutes at room temperature. Coverslips were then stained with anti-α-tubulin antibody (1:100, Genscript, cat. no. A01410-100) and DAPI (Thermo Fisher Scientific, cat. no. S36942). The mitotic index was evaluated counting the percentage of mitosis scoring at least 1000 nuclei.

***In vitro* microtubule assembly assay**

Separation of insoluble polymerized microtubules from soluble tubulin dimers was performed. Briefly, cells were treated for 48 hours with increasing concentrations of C28, 50 nM paclitaxel (Sigma Aldrich, cat. no. T1912-1MG) and 50nM colchicine (Sigma Aldrich, cat. no. C9754-100MG). Cells were then harvested and lysed using a hypotonic lysis buffer (20 mM Tris HCl pH 8, 1 mM MgCl2, 2 mM EGTA, 1% NP40), supplemented with protease and phosphatase inhibitors (Roche Diagnostics GmbH, cat. no. 11697498001 & cat. no. 4906845001). Supernatants containing the soluble fraction were collected after centrifugation at 13,000 rpm for 10 minutes at room temperature. The pellets were extracted using RIPA buffer (Sigma Aldrich, cat. no. R0278-50ML) including fresh protease and phosphatase inhibitors (Roche Diagnostics GmbH, cat. no. 11697498001 & cat. no. 4906845001). Supernatants and pellets were dissolved in SDS-PAGE sampling loading buffer at 95°C for 10 minutes and subjected to electrophoresis on SDS-polyacrylamide gels before Western blotting. The relative amounts of tubulin were detected by an anti-α-tubulin primary antibody (0.5 μg/mL, Genscript, cat. no. A01410-100) and horseradish peroxidase-conjugated secondary antibody. Chemiluminescent detection was performed using the SuperSignal West Pico PLUS Chemiluminescent Substrate (Thermo Fisher Scientific, cat. no. 34577). Emission was captured using the UVP ChemStudio Imaging Systems (Analityk jena).

***In vitro* microtubule polymerization assay**

The polymerization of purified tubulin was detected after incubation with increasing concentrations of C28 using the *in vitro* tubulin polymerization assay kit (Millipore, cat. no. 17-10194-1) according to the manufacturer’s instructions. DMSO was used as vehicle control. Nocodazole (10 μM) and paclitaxel (10 μM) were used as positive tubulin-targeting agents controls. The tubulin polymerization was measured by continuous monitoring of the turbidity change at 350 nm using the SpectraMax i3x plate reader.

**LMTK3 silencing**

Cells were transfected with a pool of 3 LMTK3 siRNAs (Ambion, cat. no. s41588, s41589 & s415890) or scramble control (Ambion, cat. no. 4390843) using the 4D-Nucleofector™ System (Lonza), following the manufacturer’s instructions. Briefly, 500,000 cells were resuspended in 20 μl of complete buffer SE (Lonza, cat. no. PBC1-00675) and 300 nM siRNA were added prior electroporation. Cells were then seeded in warm complete medium. Silencing was confirmed by western blotting.

**LMTK3 and NUSAP1 overexpression**

300,000 cells were seeded per well in a 6-well plate. Cells were then transfected with 2 μg of pCMV6-LMTK3 (Origene, cat. no. RC223140) or plasmid pCMV6-NUSAP1 (Origene, cat. no. RG219159) or pCMV6 empty vector (Origene, cat. no. PS100001) using Fugene HD transfection reagent (Promega, cat. no. E2311), following the manufacturer’s instructions. Overexpression was then confirmed by western blotting.

**Computational methods** Molecular docking calculations were performed using the X-ray crystal structure of tubulin complex with nocodazole (PDB ID: 5ca1) resolved at 2.4 Å. Protein only atoms of chains A and B were retained, then polar hydrogen atoms were added and Gasteiger charges were applied using AutoDockTools 1.5.4. The search space was centered at the colchicine-binding site of tubulin and comprised 67×67×67 grid points of 0.375 Å spacing. For each calculation, 100 docking rounds were carried out using AutoDock 4.2 and the Lamarckian genetic algorithm with default parameters, except for the maximum number of energy evaluations that was set to 10 million. The resulting conformations were clustered using a 2.0-Å cutoff, whereas visual examination of the results and rendering of the figures was performed using VMD 1.9.3. To validate the docking methodology we performed re-docking of nocodazole. The top-ranked binding pose of nocodazole displayed atomic root-mean-square deviation (RMSD) of 1.56 Å with respect to the crystallographic structure and 71% of the docked conformations within the top-ranked cluster (Δ*G*^est^ = -10.36 kcal/mol). The selected bound pose of C28 is from the top-ranked cluster comprising 63% of the docked conformations with mean estimated free energy of binding Δ*G*^est^ = -9.10 kcal/mol.

**Fluorescence measurements of C28 binding to tubulin**

The intrinsic fluorescence intensity of tubulin was measured at a QuantaMaster 400 spectrofluorimeter (Photon Technology International, Bir- mingham, NJ). Tubulin solutions of 2 μM were prepared from purified porcine tubulin (Cytoskeleton Inc., cat. no. T240) in 80 mM PIPES buffer pH 6.9, 2 mM MgCl_2_, 0.5 mM EGTA (Cytoskeleton Inc., cat. no. BST01, Cytoskeleton Inc.) and supplemented with 1 mM GTP (Cytoskeleton Inc., cat. no. BST06). Tubulin was incubated with increasing concentrations of C28 for 20 min at 25°C in 30 mm path length cells before measurement. Specifically, C28 dissolved in DMSO stock solutions of 0.5–30 mM was diluted 100-fold in the tubulin solutions, so that the final concentration of DMSO was 1% for all measurements. The fluorescence intensity was monitored in the range of 310 to 420 nm with excitation at 290 nm and without applying any correction. Fluorescence data were fitted in the following equation using GraphPrism 7.0 (Graph Pad Software, CA, USA):

$$\Delta F=\frac{{\Delta F}_{max}\times C}{K_{d}\times C}$$

ΔF is the change in the fluorescence intensity with respect to the free tubulin in buffer, ΔF_max_ is the maximum change in the intensity of tubulin, C is the concentration of the ligand C28 and *K*_d_ the apparent dissociation constant.

**TMT sample preparation**

The TMT experiment and subsequent analysis was performed by DC Biosciences (<https://dcbiosci.com/>). Stably LMTK3-overexpressing MCF7 cell pellets were lysed by resuspension of the cell pellet in 200 µL lysis buffer (PBS, 4% SDS, 25 mM TCEP supplemented with protease and phosphatase inhibitors) and sonicated for 3*5s on ice followed immediately by heating at 95°C for 10 minutes. Samples were allowed to cool down, alkylated with N-Ethylmaleimide (50 mM) in the dark for 30 min at RT, then centrifuged for 5 minutes at 17,000 g and pellets discarded. Cleared lysates were methanol-chloroform precipitated, re-dissolved in 100 µL of 0.1 M TEAB, 8 M urea, diluted 1:4 (urea to 2 M), digested at 37°C overnight with 6 µg LysC, further diluted 1:2.5 (urea 0.8 M) then digested at 37°C overnight with 6 µg Trypsin. The digestion was stopped by adding TFA (final 1%) and tryptic peptides were then desalted on a tC18 SepPak 96-well plate. Peptide concentration was determined using the Pierce Fluorometric Peptides Assay. Equal amounts of each sample were labelled with TMT-10plex, combined and fractionated (4 fractions) by High pH Reversed Phase chromatography. Peptides were cleaned-up as above and re-dissolved in 5% formic acid.

**Mass spectrometry analysis**

For MS analysis, samples were separated on an Ultimate 3000 nano-LC system coupled to an Orbitrap Fusion Tribrid mass spectrometer (Thermo Scientific). For online Reversed Phase chromatography, solvent A was 0.1% (V:V) formic acid and Solvent B was aqueous 80% acetonitrile in 0.1% formic acid. Samples were loaded onto an Acclaim PepMap C18 nano-trap column, washed with 0.5% acetonitrile 0.1%, then resolved on a 250 mm × 75 μm Acclaim PepMap C18 reversed phase analytical column (Thermo Scientific) over a 150 minutes organic gradient, using 7 gradient segments (1-6% solvent B over 1 minute, 6-15% B over 58 min, 15-32% B over 58 min, 32-40% B over 5minutes, 40-90% B over 1minute, held at 90% B for 6 minutes and then reduced to 1% B over 1 minute) with a flow rate of 300 nL/min. For MS analysis, peptides were ionized by nano-electrospray ionization at 2.0 kV using a stainless-steel emitter with an internal diameter of 30 μm (Thermo Scientific) and a capillary temperature of 275°C. All spectra were acquired using an Orbitrap Fusion Tribrid mass spectrometer controlled by Xcalibur 2.0 software (Thermo Scientific) operating in data-dependent acquisition mode. FTMS1 spectra were acquired at a resolution of 120,000 over a scan range (m/z) of 375-1550, with an AGC target of 2E5 and a max injection time of 50 milliseconds. Precursors were filtered to only include charge states 2-7. Dynamic exclusion was set to 60 seconds. Up to 12 dependent ITMS2 scans were acquired for precursors with intensities between 5000 and 1E20. The quadrupole isolation window was set to of 1.2 m/z. FirstMass was set 120, the AGC target was 1E4, max injection time was 70 milliseconds and CID collision energy was 35%. FTMS3 scans (Reagant (sic.) Tag Type set to TMT) were acquired at 50,000 resolution over a 100-500 m/z range, using a MultiNotch (5) method, HCD activation with 60% collision energy, AGC target 5E4 and 105 ms max injection time with 60% collision energy, AGC target 5E4 and 105 ms max injection time.

**Data Analysis**

Acquired raw files were searched in MaxQuant (Version 1.6.17.0) against a human database from UniProt (ID: UP000005640). The search criteria were including oxidation of methionine and acetylation of the protein N-terminus as variable modifications and N-Ethylmaleimide (NEM) modification of cysteine (+125.048) as fixed modification. For digestion mode enzyme specificity was Trypsin/P with a maximum of two missed cleavages per peptide. All FDRs were set to 1%. TMT label purity correction was performed in MaxQuant. The ‘protein groups’ table was then fed into Normalyzer (R package) for data normalisation. Quantile-normalised values were then imported into the Perseus (Version 4.1.3.0) data analysis software platform. Unpaired two sample t-tests were used to identify proteins that were differentially expressed and a secondary threshold requiring a ±1.25 Log_2_ fold change was used. The volcano plot was designed with GraphPad Prism 9.

**Co-immunoprecipitation**

Proteins were extracted and co-immunoprecipitated from 350 μg protein lysate in 150μL modified RIPA buffer [50 mM Tris-HCl pH 7.5, 150 mM NaCl, 1% sodium deoxycholate, 1% NP-40 alternative, 0.02% SDS containing protease and phosphatase inhibitors (Roche Diagnostics GmbH, cat. no.11697498001 & cat. no.4906845001)] with 1:50 anti-Flag antibody (Cell signaling, cat. no. 8146) or 1:50 anti-NUSAP1 antibody (St John’s Labs, #STJ91598) overnight at 4°C. Antigen-antibody complexes were purified using Protein G resin (Genscript, cat. no. L00209), which was pre-blocked with 1% BSA-RIPA buffer for 4 hours at 4°C. After washing of the beads, bound proteins were eluted by boiling in 1×SDS gel loading buffer and analyzed by immunoblotting.
